# Tötungsdelikte in Kliniken für Psychiatrie aus der Metaperspektive eines Bundeslandes

**DOI:** 10.1055/a-2567-7188

**Published:** 2025-06-02

**Authors:** Anke Bramesfeld, Gesa Schirrmacher

**Affiliations:** 1 Institut fur Epidemiologie, Sozialmedizin und Gesundheitssystemforschung, OE 5410, Medizinische Hochschule Hannover; 2 Abteilung 1 Soziales, Pflege & Psychiatrie, Niedersächsisches Ministerium für Soziales Arbeit Gesundheit und Gleichstellung, Hannover

**Keywords:** Psychiatrie, Tötungsdelikt, Akutstation, Patientensicherheit, Prävention, Psychiatry, Homocide, acute care unit, patient security, Prevention

## Abstract

Tötungsdelikte in Kliniken für Psychiatrie sind seltene, aber wiederkehrende Ereignisse. Anhand administrativer Meldungen werden Tötungsdelikte der Jahre 2016 bis 2024 in niedersächsischen Kliniken für Psychiatrie untersucht. 8 vollendete und 5 versuchte Tötungsdelikte wurden gemeldet. Alle erfolgten in der Erwachsenenpsychiatrie und alle bis auf eine Tat auf geschlossenen Akutstationen. In der Mehrzahl drang die Täterin/der Täter in ein fremdes Patientenzimmer ein. Unter den Opfern waren mehrere mit erhöhtem Pflegebedarf aufgrund körperlicher oder geistiger Beeinträchtigungen. Die Mehrzahl der Täter war freiwillig oder nach Betreuungsrecht untergebracht. Schlussfolgerung: Die meisten Tötungsdelikte erfolgten nicht durch Personen, die als akut fremdgefährdend eingeschätzt waren. Akutstationen sollten einen stärkeren Fokus auf den präventiven Opferschutz legen: durch die Installation von abschließbaren Patientenzimmertüren und verstärktem Schutz von körperlich oder geistig fragilen Personen mit erhöhtem Pflegebedarf.

## Abstract (en)

Homicides in psychiatric clinics are rare, however recurring incidents; using administrative data, homicides for the years 2016 to 2024 in psychiatric clinics in Lower Saxony were analysed. 8 completed and 5 attempted homicides were reported. All incidents occurred in adult psychiatry, and all but one took place in closed acute care units. In the majority of cases, the offender entered the victim's patient room. Some victims were in a fragile somatic or mental state. The majority of offenders were voluntarily or under legal guardianship hospitalized. Conclusions: Homicides in psychiatric clinics are not typically committed by patients considered acutely dangerous to others. Therefore, acute care units need to place a stronger emphasis on victim protection: Patient room doors should be lockable from the outside to prevent unauthorized access. Patients with fragile somatic and mental health conditions needing higher care should be better protected.

## Hintergrund

Versuchte und vollendete Tötungsdelikte durch oder an Patientinnen und Patienten von Kliniken der Psychiatrie sind seltene Ereignisse. Wenn überhaupt, erlebt eine Klinikleitung dies in der Regel nur ein einziges Mal in ihrer Amtstätigkeit. Der Blick aus der Metaperspektive eines Bundeslandes zeigt jedoch, dass es sich zwar um Einzelereignisse handelt, die eine absolute Ausnahme sind, die trotz alledem aber immer wieder auftreten. Das Versprechen, dass psychiatrische Krankenhäuser und insbesondere geschlossene Stationen sichere Orte sind – ein Versprechen, das wohl alle in einer Psychiatrie diensthabenden Ärztinnen und Ärzte schon einmal einer Patientin oder einem Patienten gegeben haben, und dass auch die Länder mit ihren Gesetzen über Hilfen und Schutzmaßnahmen für psychisch Kranke geben – wird durch diese Tötungsdelikte maximal konterkariert. Ziel muss es sein, die Anzahl von Tötungsdelikten in psychiatrischen Kliniken gegen null zu bringen.

Mit der Frage nach Mustern für Risikokonstellationen werden im Folgenden die Tötungsdelikte in niedersächsischen psychiatrischen Kliniken, die nach dem Niedersächsischen Gesetz über Hilfen und Schutzmaßnahmen für psychisch Kranke (NPsychKG) beliehen sind, analysiert. Aufgrund der geringen Fallzahlen ist nicht von statistischer Relevanz auszugehen, es wird jedoch deskriptiv nach Mustern für Risikokonstellationen gesucht.

## Methode

Diese Analyse berücksichtigt alle elektronisch vorhandenen Berichte über vollendete und versuchte Tötungsdelikte in psychiatrischen Kliniken , die der Fachaufsicht im Niedersächsischen Ministerium für Soziales, Arbeit, Gesundheit und Gleichstellung vorliegen.

In Niedersachsen sind 27 Kliniken der Erwachsenenpsychiatrie und 12 Kliniken der Kinder- und Jugendpsychiatrie nach dem NPsychKG beliehen. Das bedeutet, dass diese Kliniken nach Landesrecht legitimiert sind, unter bestimmten Voraussetzungen Personen auch gegen ihren Willen unterzubringen, zu sichern und zu behandeln. Eine Fachaufsicht im zuständigen Ministerium wacht darüber, dass Unterbringung, Sicherung und Behandlung gesetzeskonform erfolgt.

Die nach dem NPsychKG beliehenen Kliniken sind per Erlass verpflichtet, besondere Vorkommnisse – zu denen zweifelsohne Tötungsdelikte gehören – an die Fachaufsicht zu melden. Für diese Meldungen steht den Klinken ein standardisiertes Meldeformular zur Verfügung. Dieses ist generisch und wird auch für Meldungen zu anderen besonderen Vorkommnissen, wie z. B. Entweichungen oder sexuellen Übergriffen genutzt. Manchmal erfolgt die Meldung auch ohne die Nutzung des Formulars per formloser E-Mail. Je nach Vorkommnis und Situation folgt der Meldung ein Austausch zwischen meldender Klinik und Fachaufsicht zu weiteren Details des Vorkommnisses und eine erweiterte Berichterstattung. Dokumente dieses meist per E-Mail geführten Austausches wurden ebenfalls in die Auswertungen einbezogen.

Meldungen zu besonderen Vorkommnissen liegen der Fachaufsicht seit 2016 elektronisch vor. Die Meldungen und der Schriftverkehr zu den besonderen Vorkommnissen sind für jede der beliehenen Klinik nach Jahren hinterlegt. Diese Dateien wurden sämtlich nach Berichten über versuchte und vollendete Tötungsdelikte durchgesehen.

Als versuchtes Tötungsdelikt galten alle gemeldeten besonderen Vorkommnisse, die auch als solche von der Klinik benannt wurden. Als vollendete Tötungsdelikte galten ebenfalls die von der Klinik so benannten Vorkommnisse. Sie sind in der Regel von der Polizei auch so bestätigt worden. Die weitere juristische Einordnung der Taten durch Staatsanwaltschaften und Gerichte lässt sich den Akten der Fachaufsicht nicht entnehmen.

Die Dateien zu den gemeldeten Fällen wurden deskriptiv analysiert hinsichtlich Alter, Geschlecht, Unterbringungsstatus der Täterinnen oder Täter bzw. Alter und Geschlecht der Opfer, Ort und Art des Tötungsdeliktes sowie weiteren aus dem Schriftwechsel zwischen Fachaufsicht und Klinik extrahierbaren Informationen zur Tat.

Aufgrund der Singularität der einzelnen Taten ist die Möglichkeit gegeben, dass Personen oder Kliniken identifiziert werden können. Daher werden die Ergebnisse der Analyse und Informationen zu Hergang, Tätern, Opfern, Kliniken aggregiert dargestellt und soweit als möglich auf eine Verbindung der einzelnen Parameter verzichtet.

## Ergebnisse

In den Jahren 2016 bis August 2024 meldeten die 27 Kliniken der Erwachsenenpsychiatrie und 12 Kliniken der Kinder- und Jugendpsychiatrie 13 Vorkommnisse zu insgesamt 8 vollendeten sowie 5 versuchten Tötungsdelikten.

Sämtliche vollendete und versuchte Tötungsdelikte wurden durch Patientinnen oder Patienten der Erwachsenenpsychiatrie verübt. Alle bis auf ein Tötungsdelikt fanden innerhalb der Klinik und auf einer geschlossenen Station statt. Ein Delikt ereignete sich außerhalb der Klinik.

Die Opfer waren in der Regel Mitpatientinnen und -patienten, in einem Fall eine externe Person, in einem weiteren Fall eine Pflegeperson. Merkmale der Täterinnen und Täter, der Opfer und der Tatorte finden sich in den Tabellen 1–3 des Supplements.

- Vollendete Tötungsdelikte: Alle bis auf ein Delikt, bei dem eine externe Person getötet wurde, ereigneten sich auf geschlossenen Akutstationen. Für eines der Delikte konnte die Polizei keine Täterin oder Täter ermitteln. Die Mehrheit der bekannten Täterinnen und Täter (4) war freiwillig untergebracht. Von zwei Täterinnen oder Tätern ist der Unterbringungsstatus nicht dokumentiert, wobei bei einer oder einem von einer Unterbringung nach § 1831 BGB (bzw. § 1906 alte Fassung. BGB) ausgegangen werden kann. Alle bis auf einen der bekannten Täterinnen und Täter, hatten die Diagnose einer Erkrankung aus dem schizophrenen Formenkreis. Nur zu zwei der Täterinnen/Tätern ist im Vorfeld Gewalt gegen Dritte oder Sachen in den Berichten der Klink an die Fachaufsicht dokumentiert.

Die Tötungsarten waren Strangulation (3), Ersticken (2), Erstechen (1), stumpfe Gewalt (1) und Manipulation an einem Herzunterstützungssystem (1).

In allen Fällen von Tötungsdelikten, die in der Klinik erfolgten, fand die Tat im Zimmer der Opfer statt. Nur in einem dieser Fälle teilten sich Täterin/Täter und Opfer dasselbe Zimmer. Bei drei Opfern handelt es sich um gerontopsychiatrische Patientinnen/Patienten mit teils erheblichem Pflegebedarf. In zwei Fällen waren deren Zimmer nur schlecht vom Pflegestützpunkt aus kontrollierbar, beim dritten Fall war dies mutmaßlich so.

- Versuchte Tötungsdelikte : Auch diese ereigneten sich sämtlich auf geschlossenen Akutstationen. Die Täterinnen und Täter waren in der Mehrzahl nach § 1831 BGB (bzw. § 1906 alte Fassung BGB) untergebracht und hatten mehrheitliche eine Diagnose einer Erkrankung aus dem schizophrenen Formenkreis. Opfer waren ebenfalls Mitpatientinnen und -patienten, in einem Fall eine Pflegeperson.

Die Arten der versuchten Tötung waren in vier Fällen Strangulation und in einem eine Stichverletzung.

Alle versuchten Tötungsdelikte bis auf eines ereigneten sich in den Patientenzimmern. In zwei Fällen war die Täterin oder der Täter in das Zimmer des Opfers eingedrungen, in einem Fall teilte sie oder er das Zimmer mit dem Opfer. Ein Ereignis ereignete sich als das Opfer (Pflegeperson) sich im Zimmer des Täters aufhielt und eines ereignete sich auf den Gemeinschaftsflächen der Station.

Die Vollendung der Tötungsdelikte konnte verhindert werden, weil sich das Opfer aus dem Zimmer der Tat retten konnte, über einen Notrufsender verfügte (Pflegeperson), das Pflegepersonal zufällig dazu kam oder die Tat auf der Gemeinschaftsfläche stattfand und Mitpatientinnen und -patienten einschritten.

Wenigstens von dreien der bekannten Täterinnen und Tätern von vollendeten und versuchten Tötungsdelikten wird berichtet, dass bei ihnen eine baldige Entlassung geplant war.

## Diskussion

Die deskriptive Untersuchung von Tötungsdelikten in psychiatrischen Kliniken aus der Metaperspektive eines großen Bundeslandes zeigt, dass die Täterinnen und Täter in der Mehrheit männlich waren und an einer Schizophrenie litten. Dieses Profil ist insofern unspezifisch und entspricht der Kernklientel von geschlossenen Stationen 4 .

Der Unterbringungsstatus der Täterinnen und Täter, der mehrheitlich freiwillig oder eine Unterbringung nach BGB war, weist darauf hin, dass eine akute Fremdgefährdung eher nicht vermutete wurde. Prävention hätte also hier weniger über das Identifizieren von möglichen Täterinnen und Tätern über das übliche Gewaltscreening erfolgen können [5] als durch das Identifizieren und den Schutz möglicher Opfer.

Trotz der wenigen Fälle ergeben sich Hinweise auf mögliche Präventionsmaßnahmen:

1. Die überwiegende Mehrzahl der Delikte ereignete sich in den Patientenzimmern, und zwar mehrheitlich so, dass die Täterin oder der Täter in das Zimmer des Opfers eindrang. In allen diesen Fällen waren die Patientenzimmer nicht von außen abschließbar, sondern standen allen offen, die sie befugt oder unbefugt betreten wollten. Obwohl es technisch möglich ist, Patientenzimmer mit Schließanlagen ausrüsten, die es den Zimmerbewohnerinnen und -bewohnern und dem Klinikpersonal erlauben die Türen von außen zu öffnen, nicht aber unbefugten Personen, verfügt nur eine Minderheit der Kliniken in Niedersachsen über solche abschließbaren Zimmertüren auf Akutstationen. Von außen abschließbare Patientenzimmertüren gehören zu den Empfehlungen für eine deeskalierende bauliche Ausstattung von Akutstationen. Sie beugen nicht nur dem Eindringen unbefugter Personen vor, unabhängig von deren Motivation, sondern sie führen auch zu einem größeren Sicherheitsgefühl, Entspannung und Deeskalation auf Akutstationen 1 . Abschließbare Zimmertüren schützen allerdings nicht vor Taten durch die Zimmernachbarn. Angesichts dessen, dass die Täterinnen und Täter mehrheitlich im Vorfeld nicht als fremdgefährdend eingeschätzt wurden, ist die Unterbringung in Zweibettzimmern unter dem Aspekt des Opferschutzes kritisch zu diskutieren.
2. Die Vollendung der Tötungsdelikte konnten verhindert werden, weil das Opfer Hilfe rufen konnte oder spontan erhielt. Inwieweit die Ausstattung von Patientinnen und Patienten mit Notfallsendern eine geeignete Maßnahme zur Verbesserung der Patientensicherheit ist, sollte ebenfalls diskutiert werden.
3. Unter den Opfern der vollendeten Tötungsdelikte befindet sich eine Reihe von Personen, die eine erhöhten Pflegebedürftigkeit aufgrund körperlicher oder geistiger Beeinträchtigungen aufweisen. Diese Personen waren zum Teil auf den Stationen so untergebracht, dass eine direkte Beobachtung vom Pflegestützpunkt aus eher schwer möglich war. Es scheint wichtig zu sein, auf Akutstationen nicht nur eine Sensitivität zu haben für Personen, die ein Risiko haben fremdaggressiv zu agieren, sondern auch für Personen, die ein Risiko haben, Opfer zu werden. Dies betrifft körperlich oder geistig fragile Personen 2 3 . Diese Personen, gerade wenn sie einen erhöhten Pflegebedarf aufweisen, sollten von Akutstationen ferngehalten werden, insbesondere, wenn sie bettlägerig sind. Oder sie sollten im besonderen Beobachtungsfokus des Pflegepersonals stehen und in Sichtweite vom Pflegestützpunkt untergebracht sein.

## Limitationen

Diese Studie basiert auf Verwaltungsdaten, die aus Meldungen an die Fachaufsicht über die nach NPsychKG beliehenen Kliniken in Niedersachsen stammen. Verwaltungsdaten sind naturgemäß reduziert und auf das Nötigste beschränkt. Bei nicht-standardisierten Erfassungsprozessen können Lücken nicht ausgeschlossen werden.

Die Limitation der Verwaltungsdaten bringt es auch mit sich, dass nicht systematisch Daten zur (Über-)Belegung oder personellen Besetzung der Stationen zum Zeitpunkt der Delikte vorliegen. Anekdotisch ist von einigen Fällen bekannt, dass Überbelegung bzw. personelle Unterbesetzung eine Rolle gespielt haben mögen, doch reichen die Daten nicht, um diesbezüglich eine Aussage zu treffen.

Während bei den vollendeten Tötungsdelikten aufgrund der möglichen Medienwirksamkeit von einem vollständigen Reporting auszugehen ist, kann es bei den versuchten Tötungsdelikten zu einem Underreporting gekommen sein. Fälle, bei denen keine nach NPsychKG untergebrachten Personen involviert sind und bei denen keine Medienwirksamkeit erwartet wird, sollen, aber müssen der Fachaufsicht nicht berichtet werden. Auch können versuchte Tötungsdelikte als „normale“ Fremdaggression missinterpretiert worden sein.

Die Stärke dieser Studie liegt jedoch darin, dass sie ein Phänomen, dass zu selten ist, um es aus der einzelnen Klinik heraus zu verfolgen und über das vielleicht Fallberichte 4 , aber keine übergreifenden Daten oder Studien vorliegen, auf der Metaebene eines großen Bundeslandes untersucht.

## Schlussfolgerung

Vollendete oder versuchte Tötungsdelikte durch Patientinnen und Patienten in psychiatrischen Kliniken erfolgen nicht unbedingt durch Personen, die durch ein gewalttätiges Risikoprofil auffallen. Eine Strategie zur Vorbeugung muss daher ein stärkerer Fokus auf den Schutz möglicher Opfer sein. Dazu gehört das Installieren von Patientenzimmertüren, die von außen verschlossenen sind und nur durch befugte Personen, einschließlich der Zimmerbewohnerinnen und -bewohner zu öffnen sind und das Unterbringen in Einzelzimmern. Auch das Ausstatten von Patientinnen und Patienten mit Notfallsendern ist zu diskutieren. Ferner bedürfen Personen mit einem Opferprofil, wie körperlich oder geistig beeinträchtigte Personen mit erhöhten Pflegebedarf, einer besonderen Aufmerksamkeit auf Akutstationen. Vorzugsweise werden sie dort gar nicht untergebracht, insbesondere, wenn sie bettlägerig sind.

Die Analyse von Tötungsdelikten in Kliniken der Psychiatrie zeigt, dass Patientensicherheit nicht nur durch Identifizieren und Kontrollieren von Gewaltrisiken erreicht wird, sondern dass auch der konkrete Schutz möglicher Opfer unverzichtbar ist.

## Konsequenzen für Klinik und Praxis

- Tötungsdelikte auf psychiatrischen Akutstationen werden in der Regel nicht von Personen verübt, die als akut fremdgefährlich gelten. Zur Vorbeugung bedarf es daher nicht nur der Kontrolle möglicher Gewaltrisiken, sondern auch des Schutzes möglicher Opfer.
- Maßnahmen mögliche Opfer zu schützen umfassen von außen abschließbare Patientenzimmertüren und das Nichtunterbringen von Personen mit erhöhtem Pflegebedarf auf geschlossenen Akutstationen.
- Die Unterbringung in Einzelzimmern und die Ausstattung von Patientinnen und Patienten mit Notfallsendern zur Verbesserung der Patientensicherheit sollte diskutiert werden.

## Zusätzliches Material

Zusätzliches Material Zusätzliches Material
