# Supplementary material for: Homicides in non-forensic psychiatric clinics, seen from the perspective of a federal state
Source: Psychiatr Prax. 2025 Jun 2;52(6):332–5. [Article in German] doi: 10.1055/a-2567-7188 (PMC12440609; doi:10.1055/a-2567-7188)
Supplement: Supplementary file 1 — Zusätzliches Material [file 10-1055-a-2567-7188-pp-2025-01-0323-koa.pdf]

Zusatzmaterial

Tabelle 1 Merkmale der Täterinnen und Tätern

	Anzahl Täterinnen /Täter	Geschlecht	Alter	Diagnose	Rechtsgrundlage der Unterbringung
**Vollendetes Tötungsdelikt**	• 6<br>• 1 Person nicht ermittelt	• 5 Männer<br>• 1 Frau<br>• 1 Person unbekannt	• 21-79 Jahre alt<br>• 2 Personen Alter nicht dokumentiert<br>• 1 Person unbekannt	• 5 Personen ICD 10 F2<br>• 1 Person ICD 10 F0<br>• 1 Person unbekannt	• 4 Personen freiwillig,<br>• 2 Personen Unterbringungsstatus nicht dokumentiert<br>• 1 Person unbekannt
**Versuchtes Tötungsdelikt**	• 5	• 4 Männer<br>• 1 Frau	• 5 Personen Alter 22-55 Jahre	• 4 ICD 10 F2<br>• 1 unbekannt	• 3 Person § 1831 BGB/§ 1906 a.F. BGB,<br>• 1 Person freiwillig,<br>• 1 Person NPsychKG

Tabelle 2 Merkmale der Opfer von Tötungsdelikten

	Anzahl Opfer	Geschlecht	Alter	Besondere Merkmale der Opfer
**Vollendetes Tötungsdelikt**	• 8	• 5 Männer<br>• 3 Frauen	• 34-88, Jahre,<br>• 2 Personen nicht dokumentiert	• 3 Personen mit teils erheblicher Pflegebedürftigkeit<br>• 1 externe Person
**Versuchtes Tötungsdelikt**	• 5	• 2 Männer<br>• 3 Frauen	• 39 bis 68 Jahre<br>• 2 Personen nicht dokumentiert	• 1 Person mit geistiger Behinderung<br>• 1 Pflegeperson

Psychiat Prax |10.1055/a-2567-7188| © 2025. The Author(s).

Tabelle 3 Merkmale des Tatortes

	**Tatort (gezählt pro Delikt)**	**Tatort Patientenzimmer des Opfers (gezählt pro Delikt)**	**Tatort Patientenzimmer der Täterin/des Täters**	**Anderer Tatort**
**Vollendetes Tötungsdelikt**	• 7 geschlossene Akutstation<br>• 1 außerhalb der Klinik	• 6	• 1	• 1 Privatwohnung
**Versuchtes Tötungsdelikt**	• 5 geschlossene Akutstation	• 2	• 2	• 1 Speiseraum

Psychiat Prax |10.1055/a-2567-7188| © 2025. The Author(s).
